# Effect of Selenium on the Responses Induced by Heat Stress in Plant Cell Cultures

**DOI:** 10.3390/plants7030064

**Published:** 2018-08-11

**Authors:** Massimo Malerba, Raffaella Cerana

**Affiliations:** 1 Dipartimento di Biotecnologie e Bioscienze, Università degli Studi di Milano-Bicocca, 20126 Milan, Italy; massimo.malerba@unimib.it; 2 Dipartimento di Scienze dell’Ambiente e della Terra, Università degli Studi di Milano-Bicocca, 20126 Milan, Italy

**Keywords:** cell death, heat stress, plant cell cultures, selenium, tobacco BY-2

## Abstract

High temperatures are a significant stress factor for plants. In fact, many biochemical reactions involved in growth and development are sensitive to temperature. In particular, heat stress (HS) represents a severe issue for plant productivity and strategies to obtain high yields under this condition are important goals in agriculture. While selenium (Se) is a nutrient for humans and animals, its role as a plant micronutrient is still questioned. Se can prevent several abiotic stresses (drought, heat, UV, salinity, heavy metals), but the action mechanisms are poorly understood. Se seems to regulate reactive oxygen species (ROS) and to inhibit heavy metals transport. In addition, it has been demonstrated that Se is essential for a correct integrity of cell membranes and chloroplasts, especially the photosynthetic apparatus. Previous results showed that in tobacco (*Nicotiana tabacum* cv. Bright-Yellow 2) cultures HS (5 min at 50 °C) induced cell death with apoptotic features, accompanied by oxidative stress and changes in the levels of stress-related proteins. In this work we investigated the effect of Se on the responses induced by HS. The obtained results show that Se markedly reduces the effects of HS on cell vitality, cytoplasmic shrinkage, superoxide anion production, membrane lipids peroxidation, activity of caspase-3-like proteases, and the levels of some stress-related proteins (Hsp90, BiP, 14-3-3s, cytochrome *c*).

## 1. Introduction

Plants are heterothermic sessile organisms in thermal equilibrium with the environment. Strong temperature variations exceeding lower or higher limits of the thermal optimum for the life of the plant are sensed as thermal stress, cold stress or heat stress (HS), respectively. Thermal stress can compromise the vital functions of the plant more or less severely, depending on the organ affected and its developmental stage [1]. In fact, roots may tolerate lower temperatures than stems and expanded leaves tolerate higher temperatures than the young ones. Thermal stress, in particular HS is one of the main causes of the reduction in crops productivity because the light energy required for photosynthesis results in a considerable increase of the temperature in the exposed tissues [2]. In addition, HS is able to influence growth and biodiversity of forests [3]. This is very important considering the global warming in progress in present years with increasing temperatures and decreasing precipitation with a consequent reduction of wetlands and an increase in areas at risk of desertification [3]. In fact, forests, thanks to their ability to fix carbon dioxide, absorb greenhouse gases and filter anthropogenic pollutants, potentially play a crucial role in the moderation of these changes [3]. Thus, the study of the effects of HS is of great interest for plant biologists.

Plants have evolved different responses to HS to minimize damage and ensure the conservation of cell homeostasis. An intense HS causes a “Heat shock response” (Hsr), which involves the rapid activation of “HS genes” due to the specific transcription factors, named “HS factors” (Hsf). The activity of Hsf induces the synthesis of specific “Heat shock proteins” (Hsp), that act as molecular chaperones involved in plant tolerance to a wide range of stresses [2]. HS can lead to protein denaturation and alteration of the membrane fluidity. This effect may result in high production and accumulation of reactive oxygen species (ROS) causing oxidative stress and, hence, cellular necrosis. On the other hand, low ROS concentrations may act as a second messenger for signal transduction pathways regulating a wide range of cellular functions including programmed cell death (PCD) [4,5]. PCD is an ubiquitous genetically controlled process aimed at eliminating cells that are not necessary or harmful for the proper development of the organism. Among the others, the form of PCD that is the object of major studies and is therefore better known, is the apoptosis of animal cells. Various forms of PCD are also observed in plants, where they are induced by various biotic and abiotic stimuli, including HS [6]. For a long time, selenium (Se) was considered toxic until it was recognized as a micronutrient for humans and other animals [7]. Se is present as selenocysteine in the catalytic site of several selenoproteins involved in important metabolic processes, such as thyroid hormone metabolism, mechanisms of protection from oxidative stress and immune response [8]. In several countries the very low soil concentration of Se causes deficiency in the diet of more than a billion people worldwide [9]. This implies important health problems [10]. Cultivated plants are an important source of Se for humans and livestock. Being chemically analogous to sulphur, Se is absorbed by all plants by sulphate transporters and is sequestered in the form of selenite and selenate [11]. The levels of Se accumulation depend on the abundance of Se in the soil and the levels of the sulphur compounds that compete for absorption [12]. Several attempts were made in order to increase Se content in plants. Changes in the enzymes associated with sulphur metabolism have been widely used to vary Se levels in plants [13]. Recent researches use plant-microbome interactions to increase biofortification with Se and cultivate accumulating plants on seleniferous soils, thus ameliorating soil characteristics for further cultivation. In addition, the biomass of these accumulating plants could be used to enrich the diet of people and their livestock. Finally, given that different species of plants seem able to affect the accumulation of Se from nearby plants and perhaps even their speciation, different co-cultivation techniques could be tested to optimize biofortification with Se of the cultivated plants and their nutritional quality [14]. Despite these studies, until now, there has been no clear evidence of Se essentiality for plants growth. The metal seems to play a dual role: At high doses, it acts as a pro-oxidant agent, causing serious damage to the plant, while low doses can counteract abiotic stress induced by high temperatures, drought, intense light, UV rays, excess of water, salinity and heavy metals [15]. The accumulation of ROS in response to excess of Se may depend on an insufficient presence of antioxidant compounds such as reduced glutathione, thiols, reduced ferredoxin and/or NADPH [16]. These compounds are also involved in the assimilation of Se, thus their concentration can be insufficient to satisfy necessity for Se uptake and at the same time to contrast accumulation of ROS [16]. In contrast, low levels of Se can decrease accumulation of ROS, especially O_2_^.−^ and/or H_2_O_2_, in plants subject to different stresses. Reduction of O_2_^.−^ levels can depend on: spontaneous dismutation of O_2_^.−^ to H_2_O_2_ (not catalyzed by the enzyme superoxide dismutase SOD) [17], direct elimination of O_2_^.−^ by Se compounds [18], regulation of antioxidant enzymes [15]. However, the mechanisms associated with the protective effect of Se against stresses appears complex and not yet fully understood. In addition to involvement in the mechanism of ROS regulation, a role for Se has been proposed in the inhibition of absorption and translocation of heavy metals. Furthermore, it seems to play a fundamental role in the reconstitution of the cell structures and chloroplasts, and in the recovery of the photosynthetic apparatus after stress [15]. However, an excess of Se could exacerbate the damage to the photosynthetic apparatus and could result in overproduction of starch [19]. 

Cultured cells are a good experimental material to investigate the responses elicited by HS due to their greater homogeneity compared to complex tissues. Furthermore, this system can be more controlled thus increasing the reproducibility of stress conditions. Previous results showed that in tobacco (*Nicotiana tabacum* cv. Bright-Yellow 2) cultures HS (5 min at 50 °C) induced cell death with apoptotic features, accompanied by oxidative stress and changes in the levels of stress-related proteins [4,6]. In this work we investigated the effect of Se on the responses induced by HS. The obtained results show that Se markedly reduces the effects of HS on cell vitality, cytoplasmic shrinkage, superoxide anion production, membrane lipids peroxidation, activity of caspase-3-like proteases, and the levels of some stress-related proteins (Hsp90, BiP, 14-3-3s, cytochrome *c*).

## 2. Results 

### 2.1. HS and Se Effects on Cell Viability and Cytoplasmic Shrinkage

**Figure 1:**
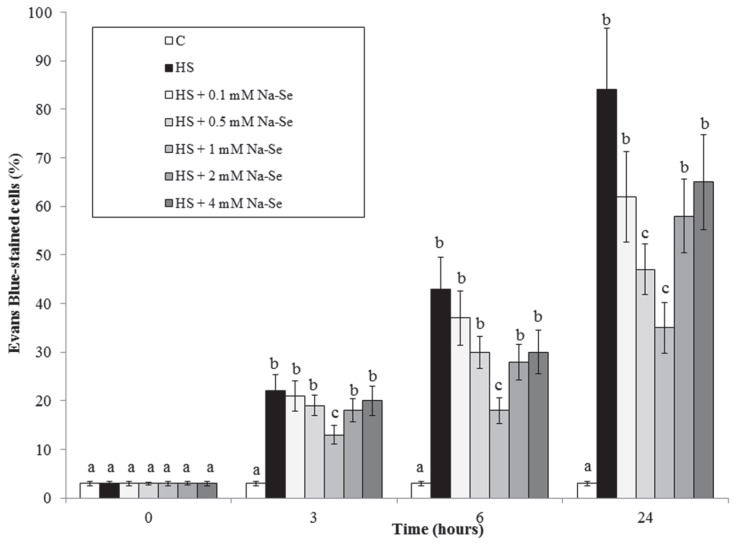
Effect of different Se concentrations on HS-elicited accumulation of dead cells in the cultures. Means ± SD (n ≥ 9) are shown. Different letters show significant differences among treatments at each time (Tukey HSD test, *p* ≤ 0.05).

To our knowledge, the effect of Se on plant cultured cells has never been investigated. Thus, to identify the most appropriate Se concentration to use in subsequent experiments, in preliminary experiments we evaluated the effects of different Se concentrations on the accumulation of dead cells induced by HS. Figure 1 shows that in cell cultures not subjected to HS, the percentage of dead cells is very low and does not vary during the experiment. HS determines a progressive accumulation of dead cells, already evident after 3 h of treatment. The results show that there is a progressive protective effect on the appearance of dead cells induced by HS by increasing the concentration of Se up to 1 mM. A further increase in Se concentration does not ameliorate the protective effect but rather seems to reduce it. Therefore, the concentration of 1 mM Se was used in subsequent experiments.

To better characterize the process of death induced by HS, we considered the appearance of cells with shrinked cytoplasm. This morphological modification is presumably caused by destructuration of the cytoskeleton, and in cultured cells is considered an index of PCD with apoptotic features [4,20].

**Figure 2:**
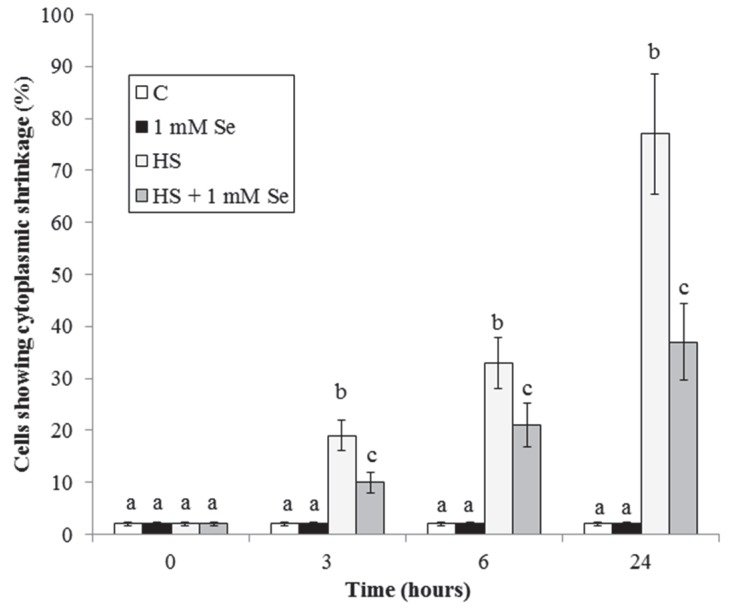
Effect of Se on HS-elicited cytoplasmic shrinkage. Means ± SD (n ≥ 9) are shown. Different letters show significant differences among treatments at each time (Tukey HSD test, *p* ≤ 0.05).

Figure 2 shows that, similarly to the percentage of dead cells, the percentage of control cells with shrinked cytoplasm is very low, constant over time and not influenced by the presence of Se. The treatment with HS leads to a considerable increase in the percentage of cells with shrinked cytoplasm that is in part prevented by Se. At all experimental times it is observed that the percentage of dead cells is slightly higher than that of cells with shrinked cytoplasm, suggesting the presence of different forms of cell death [21].

### 2.2. HS and Se Effects on Accumulation of O_2_^.−^ and MDA and on Caspase-3-like Activity

**Figure 3:**
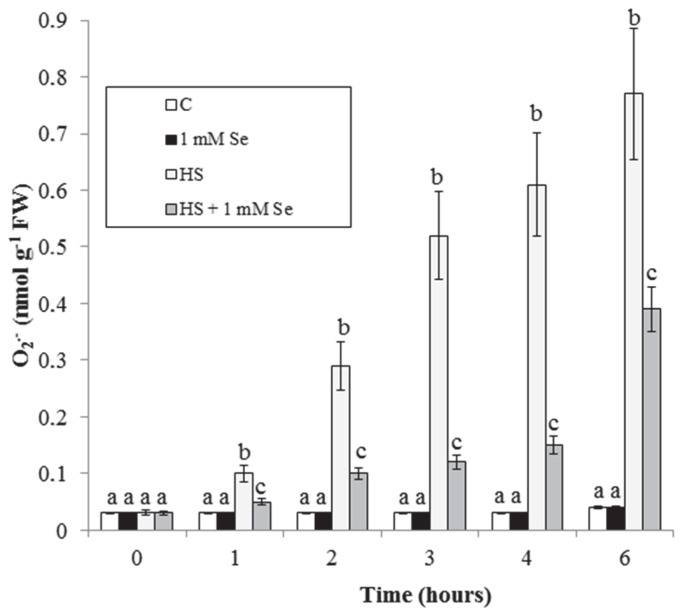
HS and Se effects on O^2−^ accumulation in the culture medium. Means ± SD (n ≥ 9) are shown. Different letters show significant differences among treatments at each time (Tukey HSD test, *p* ≤ 0.05).

O_2_^.−^ is a highly reactive ROS, responsible for important oxidative damage [21]. Treatment with HS (Figure 3) causes a progressive accumulation of O_2_^.−^. This accumulation is almost totally prevented by Se, at least for the first experimental times (up to 4 h).

**Figure 4:**
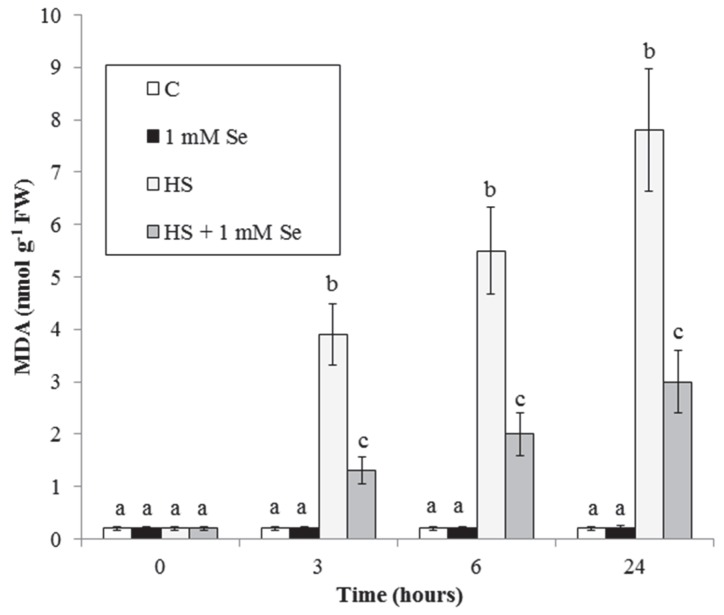
HS and Se effects on MDA accumulation. Means ± SD (n ≥ 9) are shown. Different letters show significant differences among treatments at each time (Tukey HSD test, *p* ≤ 0.05).

At cell level, peroxidation of membrane lipids is one of the main damages induced by oxidative stress and its degree was assessed by determining the level of malondialdehyde (MDA), a byproduct of polyunsaturated fatty acids oxidation, which typically originates after oxidative stress [21]. Figure 4 shows that the level of MDA of the control cells is low, constant, and not influenced by Se. At each time, HS causes a considerable production of MDA, significantly decreased by Se.

**Figure 5:**
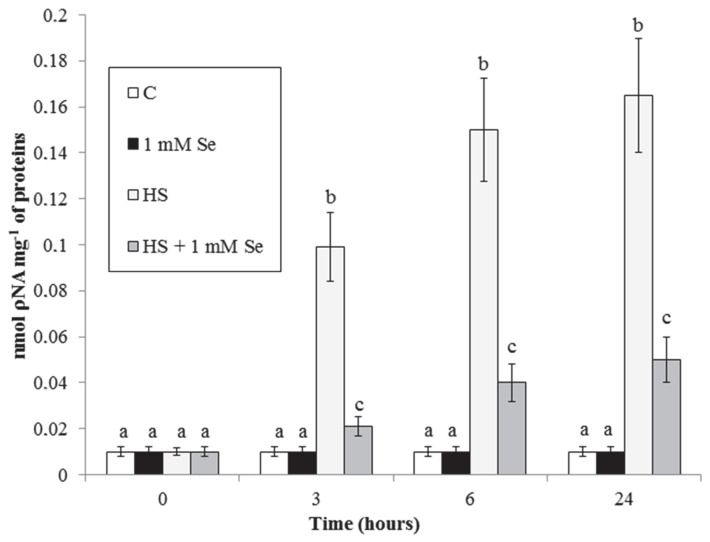
HS and Se effects on caspase-3-like activity. Means ± SD (n ≥ 6) are shown. Different letters show significant differences among treatments at each time (Tukey HSD test, *p* ≤ 0.05).

To characterize further the effect of Se on HS-elicited cell death we analysed caspase3-like proteases activity, another typical PCD marker that often increases during plant PCD. Figure 5 shows that Se strongly reduces the HS-elicited marked increase in this activity.

### 2.3. HS and Se Effects on Stress-related Proteins

Finally, we analysed the effect of HS and Se on the levels of some stress-related proteins by gel blotting. Mitochondrial Hsp 90 are molecular chaperones that control the activity of different substrates. BiP, an Hsp70 present in the endoplasmic reticulum, accumulates under different stress conditions. The regulatory proteins 14-3-3s control many processes of plant cells, including cell death and cytochrome *c* release from the mitochondrion to the cytosol, a marker of apoptotic death in animals and plants [21].

**Figure 6:**
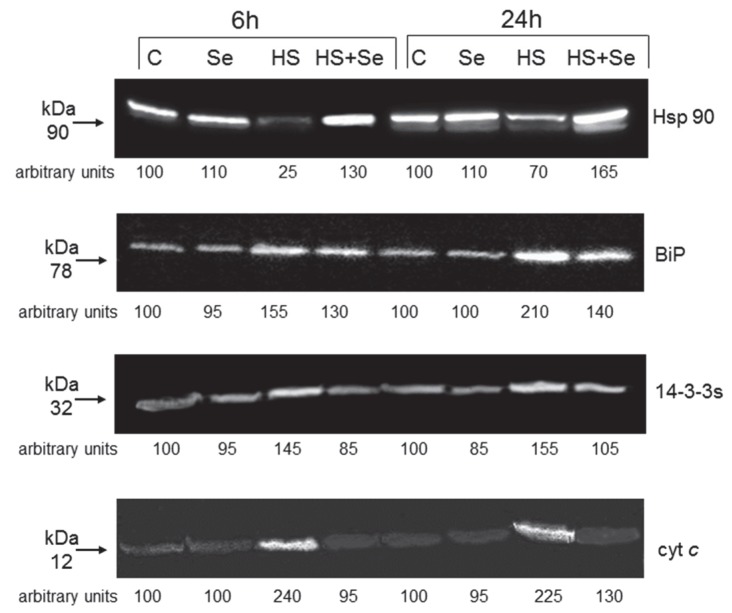
HS and Se effects on the levels of stress-related proteins. (C) control; (Se) cells + 1 mM Se; (HS) HS-treated cells; (HS + Se) HS-treated cells + 1 mM Se. Results of a typical experiment (n = 3) run in duplicate are presented. 50 mg of proteins were run in each lane. An arbitrary value of 100 was assigned to the quantity of immunodecorated protein of the controls.

Figure 6 confirms the previously reported effects of HS on the examined proteins [21], and shows that at both times Se diminishes the accumulation of microsomal BiP and almost completely prevents the accumulation of cytosolic 14-3-3s, the reduction of mitochondrial Hsp90 and the release of cytochrome *c* from mitochondria elicited by HS.

## 3. Discussion

The influence of selenium on the HS-elicited responses of tobacco cells was tested by measuring in the absence and presence of Na-selenate the following parameters: cell viability, cytoplasmic shrinkage, superoxide anion production, membrane lipids peroxidation, activity of caspase-3-like proteases, and the levels of some stress-related proteins (Hsp90, BiP, 14-3-3s, cytochrome *c*).

### 3.1. HS and Se Effects on Cell Viability and Cytoplasmic Shrinkage.

This work (Figure 1 and Figure 2) shows that Se strongly reduces the previously reported eliciting effect of HS on cell death and cytoplasmic shrinkage [6].

At proper concentrations, Se promotes growth, delays plant senescence, and precocious fruit ripening induced by different abiotic stresses, HS included [13]. Leaf and fruit senescence are processes involving programmed cell death, and when not precisely regulated can lead to important decreases in productivity in several horticultural species. The protective effect of Se against inappropriate senescence could be due to its reported ability to reduce respiratory intensity and ethylene production in different plant species [13]. Interestingly, the inhibitor of ethylene production Co^2+^ prevents cell death and cytoplasmic shrinkage induced by HS in tobacco cell cultures [6].

### 3.2. HS and Se Effects on O_2_^−.^ and Malondialdehyde Accumulations.

The HS-elicited oxidative stress with accumulation of O_2_^.−^ and malondialdehyde is largely inhibited by Se (Figure 3 and Figure 4). Similar results have been recently obtained in cucumber plants under HS and in *Zea mays* exposed to water stress where stress-induced accumulations of O_2_^.−^ and MDA are prevented by Se [22,23]. These results are not surprizing. In fact, Se acts as an antioxidant in different plant species under biotic and abiotic stresses. This protective effect of Se depends on the induced higher activity of several antioxidant enzymes and on the increased content of some antioxidant compounds (glutathione and flavonoids) [11,13].

### 3.3. HS and Se Effects on Caspase-3-like Activity and on Cytochrome c Release

Specific cysteine proteases named caspases are required for the progression of animal apoptosis. In plant cells too there are proteins with similar activity called caspases-like or metacaspases [6]. Se (Figure 5) largely prevents the HS-elicited increase in the activity of these enzymes, reported in our previous work [21]. Another ubiquitous marker of apoptotic-like PCD related to caspases is the release of cytochrome *c* from the mitochondrion [6]. We previously reported induction of cytochrome *c* release by HS [6,21]. Here we show that this release is markedly reduced by Se (Figure 6). These protective effects of Se can be due to its antioxidant effect (ROS are potent regulators of PCD) or to the effect of Se on expression of genes implied in antioxidant activity and defense responses ([11] and see below for further discussion). 

### 3.4. HS and Se Effects on the Levels of Hsp90, BiP and 14-3-3s.

As widely reported, plants evolved a set of responses to deal with HS, that includes changes of biochemical and physiological processes due to modifications of gene expression. These modifications can result in acclimation or adaptation to stress [24]. In this investigation, we studied the HS and Se effects on some stress-related proteins. Our data confirm the previously reported effects of HS on the examined proteins [21] and show that Se diminishes the accumulation of BiP and almost completely prevents the accumulation of 14-3-3s and the reduction of Hsp90 elicited by HS (Figure 6).

To our knowledge, a genomic approach has scarcely been used to study the protective role of Se against stresses. Sun and co-workers performed a comparative proteomics analysis on cucumber plants treated with Cd [25]. Comparing 2-DE gels, these researchers observed several protein spots changed by Se+Cd compared to Cd alone. By MALDI–TOF–MS mass spectrometry, they identified proteins whose relative abundance was significantly reduced by Cd and restored by Se. Among the others, ascorbate oxidase, glutathione-S-transferase and Hsp STI-like expression were strongly reduced by Cd and reincreased by Se [25]. More studies were conducted on the Se effect on the proteome of different plant species. For example, Wang and co-workers by 2-DE gels and MALDI-TOF/TOF mass spectrometry performed a comparative proteomics analysis on the effect of different Se concentrations on rice seedlings [19]. Their results showed that low (non-toxic) Se concentrations up-regulate proteins involved in ROS detoxification and resistance to pathogens such as beta-1,3-glucanase and chitinase. The expression of the same proteins was down-regulated by high (toxic) Se concentrations [19]. Finally, the Se hyperaccumulator *Stanleya pinnata* shows higher expression of genes involved in sulphur uptake and assimilation, antioxidant activities and defense compared to the related secondary Se accumulator *Stanleya albescens* [26]. In particular, the hyperaccumulator species shows a higher expression than the related species for genes encoding Hsp and luminal chaperones such as BiP both in the absence and in the presence of Se [26].

## 4. Material and Methods

### 4.1. Cell Culture Growth and Experimental Conditions 

Growth of tobacco BY-2 (*Nicotiana tabacum* L. cv Bright-Yellow 2) cells and heat treatment (5 min at 50° C) were performed as described [6]. Na-selenate (Na_2_SeO_4_) was supplied 10 min before HS.

### 4.2. Cell Death and Cytoplasmic Shrinkage Assays

Cell death was estimated spectrophotometrically with the vital dye Evans Blue as in Reference [21]. The percentage of cells showing cytoplasmic shrinkage was determined as in Reference [6]. 

### 4.3. O_2_^.−^ Assay 

The O_2_^.−^ anion generation was evaluated spectrophotometrically as reduction of XTT to XTT formazan as described [21]. 

### 4.4. Proteases Activity and Membrane Lipid Peroxidation

Caspase3-like proteases activity was measured spectrophotometrically with a caspase-3 colorimetric activity assay kit following the manufacturer’s instructions (BioVision Research Products, Mountain View, CA 94043, USA) as described [21].

The level of membrane lipid peroxidation was evaluated spectrophotometrically by measuring the content of malondialdehyde, a secondary end product of the oxidation of polyunsaturated fatty acids [21].

### 4.5. SDS-PAGE and Protein Gel Blots

Cells were collected by gentle centrifugation, frozen in liquid nitrogen and homogenized for 5 min at maximum speed with a Ultra-Turrax T25 device. The cell homogenate was differentially centrifuged to obtain the different fractions (i.e., mitochondrial, microsomal and soluble) for SDS-PAGE analysis as described [21].

Equal amounts of proteins were separated by discontinuous SDS-PAGE (4% stacking, 10% resolving gel) as described [21]. Immunodecorations of cytochrome *c*, 14-3-3 proteins, Hsp 90 and BiP were perfomed as described [21].

### 4.6. Statistical Analyses

GraphPad Prism 4 program from GraphPad Software, Inc., San Diego, CA, USA was used to statistically analyse the results. Tukey HSD test, *p* ≤ 0.05, was used in the study.

## 5. Conclusions

To summarize, Se can effectively reduce the effect of HS on cell death with apoptotic features, oxidative stress and levels of stress-related proteins. This protective effect of Se can be due to its direct antioxidant effect and/or to an effect on the expression of genes implied in antioxidant activity and defense responses. In the future, molecular and genomic studies could be valuable to elucidate the mechanisms associated with the protective effect of Se against heat stress in cell cultures as well as in plants.
